# A transdiagnostic approach to negative symptoms: exploring factor structure and negative symptoms in bipolar disorders

**DOI:** 10.3389/fpsyt.2023.1136097

**Published:** 2023-06-16

**Authors:** Henrik Myhre Ihler, Siv Hege Lyngstad, Lynn Egeland Mørch-Johnsen, Trine Vik Lagerberg, Ingrid Melle, Kristin Lie Romm

**Affiliations:** ^1^Norment, Division of Mental Health and Addiction, Oslo University Hospital, Institute of Clinical Medicine, University of Oslo, Oslo, Norway; ^2^Nydalen DPS, Division of Mental Health and Addiction, Oslo University Hospital, Oslo, Norway; ^3^Department of Psychiatry and Department of Clinical Research, Østfold Hospital, Grålum, Norway; ^4^Early Intervention in Psychosis Advisory Unit for South-East Norway, Division of Mental Health and Addiction, Oslo University Hospital, Oslo, Norway

**Keywords:** bipolar disorder, negative symptoms, diminished expression, apathy, cannabis use

## Abstract

**Background:**

Negative symptoms are increasingly recognized as transdiagnostic phenomena, linked to reduced quality of life and functioning, and often caused or worsened by amendable external factors such as depression, social deprivation, side-effects of antipsychotics or substance use. The structure of negative symptoms fits into two dimensions: diminished expression and apathy. These may differ in association with external factors that influence their severity and may thus require different treatment approaches. The dimensions are comprehensively established in non-affective psychotic disorders but are understudied in bipolar disorders.

**Methods:**

We conducted exploratory and confirmatory factor analyses in a sample of 584 individuals with bipolar disorder to assess the latent factor structure of negative symptoms as measured by the Positive and Negative Syndrome Scale (PANSS), and performed correlational analyses and multiple hierarchical regression analyses to investigate links between the two dimensions of negative symptoms and clinical and sociodemographic correlates.

**Results:**

The latent factor structure of negative symptoms fits into two dimensions, i.e., diminished expression and apathy. A diagnosis of bipolar type I or a history of psychotic episodes predicted more severe levels of diminished expression. Depressive symptoms were associated with more severe negative symptoms across dimensions, yet 26.3% of euthymic individuals still displayed at least one mild or more severe negative symptom (PANSS score ≥ 3).

**Discussion:**

The two-dimensional structure of negative symptoms seen in non-affective psychotic disorders reproduces in bipolar disorders indicating similarities in their phenomenology. Diminished expression was associated with a history of psychotic episodes and a diagnosis of BD-I, which may infer closer connections to psychosis liability. We found significantly less severe negative symptoms in euthymic than depressed participants. Nevertheless, more than a quarter of the euthymic individuals had at least one mild negative symptom, demonstrating some degree of persistence beyond depressed states.

## 1. Introduction

Negative symptoms have been considered intrinsic to psychotic disorders and are part of the diagnostic criteria for schizophrenia spectrum disorders (SCZ), i.e., schizophrenia, schizophreniform disorder and schizoaffective disorder (1–3). The underlying neurobiological mechanisms of negative symptoms are largely unknown. Still, they are of increasing interest (4, 5), due to the strong associations with low remission rates, reduced quality of life and impaired functioning (6, 7). Recent developments in psychiatry have moved toward a dimensional rather than a categorical understanding of clinical symptoms and diagnoses (8, 9). Negative symptoms are thus not pathognomonic to any specific psychiatric or neurological disorder – despite their historical link to schizophrenia – and are regularly present in other psychiatric disorders, such as bipolar disorder (BD) (10). Previous estimates of negative symptoms’ prevalence vary significantly across diagnostic categories (10), and range from rare in healthy adults (11), to 20–89% in affective disorders, and 32–80% in neurological disorders (12), yet little is still known about prevalence and characteristics in subcategories of BD.

BD is usually categorized as an affective disorder (1–3), even if genetic, neuroimaging and clinical studies find significant overlaps with SCZ (13–16). As SCZ, BD is considered a severe mental disorder, associated with impairments in cognition and functioning, and a reduced quality of life (17–19). Furthermore, severe mental disorders share environmental risk factors including substance use, adverse life events, and early trauma (20, 21). Development into different clinical phenotypes may be based on individual combinations of these factors (20, 22). Positive psychotic symptoms are present in up to 70% of individuals with BD type I (BD-I) (23, 24). BD is thus often considered part of the psychosis spectrum continuum (23, 25). One problem with diagnostic categories is the potential to ignore or misinterpret relevant symptoms that occur across a diagnostic spectrum because the symptoms are seen as a criterion for a specific diagnosis (e.g., negative symptoms and schizophrenia). The presence of negative symptoms in other disorders may thus be overlooked. Indeed, a recent study found that euthymic participants with BD-I had equal levels of apathy as participants with SCZ (26). Yet, negative symptoms are understudied in BD. This limits our progress in understanding these phenomena, and hampers the development and provision of proper treatment.

In SCZ, negative symptoms comprise two main dimensions, i.e., diminished expression (blunted affect and alogia, also called expressive negative symptoms) and apathy (avolition, anhedonia and asociality, also called experiential negative symptoms) (27, 28). This distinction is important as it could mirror different underlying pathophysiological mechanisms (4). Previous research has, however, mainly focused on negative symptoms as a unidimensional construct, which limits the understanding of diminished expression and apathy as potential separate entities. Exploratory and confirmatory factor analyses have shown that the two dimensions can be assessed using the Positive and Negative Syndrome Scale (PANSS) (29–32). However, these studies have only included participants with SCZ, and there is limited knowledge about the robustness of the two-dimensional structure in other severe mental disorders such as BD.

One way to approach this issue is to study whether current knowledge about negative symptoms in SCZ, such as their dimensional structure, translates across diagnostic groups. Two previous studies have investigated and compared the structure of psychotic symptoms in SCZ and BD using the PANSS (33, 34). Both studies employed a five-factor model of the PANSS scores and they replicated unidimensional negative symptoms as one factor in BD. The negative symptom factor could be distinguished from the depressive factor in both studies, in line with findings in SCZ and other non-affective psychoses (35–38). Differentiating negative from depressive symptom dimensions is clinically challenging (39) because of similar clinical presentations. Neither of these two studies, however, used a two-dimensional measure of negative symptoms, nor investigated clinical and sociodemographic correlates of the identified negative factor.

Strauss et al. (40) examined the two-dimensional structure of negative symptoms using the Brief Negative Symptom Scale (BNSS) to investigate symptom levels and internal consistency in SCZ, BD and healthy controls (HC). They found higher levels of the two negative symptom dimensions in both SCZ and BD, compared to HC, in addition to good internal consistency. Discriminant validity was assessed with correlational analyses to other symptom dimensions and external validators. In BD, the total score of negative symptoms and apathy was significantly correlated with depressive symptoms as measured by the Hamilton Depressive Rating Scale (HDRS) (*r* = 0.38 and *r* = 0.53, respectively), while diminished expression was not. Apathy was also significantly correlated with positive symptoms (*r* = 0.37). In addition, both dimensions were significantly correlated with the use of antipsychotics. The authors concluded that this might indicate that the negative symptoms they assessed in BD could be secondary to depression, psychotic symptoms or antipsychotic effects, a phenomenon also seen in SCZ and other non-affective psychoses (5, 39).

Another important factor associated with negative symptoms is substance use (39). We recently demonstrated an association between cannabis use and diminished expression in SCZ (41). Intake of cannabis can cause acute psychiatric symptoms (42), and cannabis use is further associated with increased risk of several detrimental outcomes (43, 44). Importantly, almost a quarter of patients with BD either use cannabis or have a cannabis use disorder (CUD) (44).

In sum, it is important to investigate the dimensional structure of negative symptoms, and their sociodemographic and clinical correlates, transdiagnostically. It may progress our understanding of these debilitating phenomena, and ultimately pave the way for new treatment approaches. The aims of the current study were:

- To explore if the two-factor structure of negative symptoms previously shown in SCZ can be replicated in a sample of BD participants.
- To validate the findings by examining the association between the identified factors and clinical characteristics previously found to be associated with negative symptoms in SCZ, including cannabis use.
- To characterize the prevalence of negative symptoms in BD, across diagnostic subgroups and affective states.

## 2. Materials and methods

### 2.1. Study design and sample

The current study is part of the ongoing Thematically Organized Psychosis study (TOP) at the Norwegian Centre for Mental Disorders Research (NORMENT). Information about the TOP-study and research activity at NORMENT were disseminated to health care professionals working in the in- and outpatient mental health care services in the Oslo catchment area through on-site visits by our research personnel and website information. Additionally, user representatives from the Norwegian Bipolar Association disseminated information to their members. Participants with an established or suspected diagnosis of BD were then recruited by referrals from the treating specialist or GP. The inclusion criteria were: age 18–65 years; meeting DSM-IV criteria (1) for BD-I, BD-II or BD not otherwise specified (BD-NOS), speaking and understanding a Scandinavian language and being able to give a written, informed consent. Exclusion criteria were IQ below 70, a history of moderate or severe head injury, somatic illness or neurological disorder that could influence or mimic affective states.

A total sample of 584 participants with BD (BD type 1: *n* = 348, BD type 2: *n* = 201, BD NOS = 35) was included for analysis. Thirty-eight participants had missing data on affective state, and were excluded from analyses that included these variables.

### 2.2. Clinical assessment

Trained clinical research personnel (medical doctors or psychologists) undertook diagnostic interviews with the Structured Clinical Interview for DSM-IV Axis I Disorders (SCID-I) (45), including the modules for affective and psychotic disorders, and substance use disorders. Detailed sociodemographic data were collected by interview and self-report. They included the history of previous and current frequency of alcohol-, nicotine-, cannabis- and other illegal substance use, and information regarding current psychotropic medication use. Premorbid social and academic functioning was assessed with the Premorbid Adjustment Scale (PAS) (46), and expressed by the sum score of PAS childhood and early adolescence for both domains. Current symptoms were assessed with the following scales: psychotic symptoms with the Positive and Negative Syndrome Scale (PANSS) (47), depressive symptoms with the Inventory for Depressive Symptomatology (IDS) (48), manic symptoms with the Young Mania Rating Scale (YMRS) (49). Positive symptoms were expressed as the sum score of PANSS items P1, P3, P5 and G9 (35). Euthymia was defined as IDS < 12 (i.e., no or mild depressive symptoms) and YMRS <8 (i.e., no or mild (hypo-)manic symptoms). Potential side-effects of medications were assessed with the Udvalg for Kliniske Undersøgelser (UKU) side-effect rating scale (50). Relevant side-effects for the current analyses were rated as present if an UKU scale score of ≥2 for the items: 1.10 emotional indifference, 2.1 dystonia, 2.1 rigidity, and 2.3 hypokinesia/akinesia.

### 2.3. Selecting the specific items for each negative symptom dimension

While several studies have validated a two-factor model of negative symptoms in PANSS in SCZ, the specific items included in each factor differ slightly (29–31, 51). The recent review by The European Psychiatric Association (EPA) (28) also underlines that some items from the general psychopathology subscale show more instability. For these reasons, we chose to investigate three possible two-factor solutions, and one unidimensional solution, as displayed in Table 1. Negative symptom scores were operationalized as the sum of included items, divided by the total number of items included.

**Table 1:** Possible factor solutions for negative symptoms in PANSS.

Solution 1 (29, 30)		Solution 2 (31, 32)		Solution 3 (28)		Solution 4
Dim. Exp.	Apathy	Dim. Exp.	Apathy	Dim. Exp.	Apathy	Unidimensional
N1 Blunted affect	N2 Emotional withdrawal	N1 Blunted affect	N2 Emotional withdrawal	N1 Blunted affect	N2 Emotional withdrawal	N1 Blunted affect
N3 Poor rapport	N4 Apathetic social withdrawal	N3 Poor rapport	N4 Apathetic social withdrawal	N3 Poor rapport	N4 Apathetic social withdrawal	N2 Emotional withdrawal
N6 Lack of flow	G16 Active social avoidance	N6 Lack of flow	G16 Active social avoidance	N6 Lack of flow		N3 Poor rapport
G5 Mannerisms and posturing		G7 Motor retardation				N4 Apathetic social withdrawal
G7 Motor retardation						N6 Lack of flow
G13 Avolition						

Dim. Exp., diminished expression; PANSS, the positive and negative syndrome scale.

### 2.4. Statistics

IBM SPSS package 27 and STATA SE 16 were used for data analyses.

First, we did an Exploratory Factor Analysis (EFA) (with Principal Axis Factoring) of the presupposed two-factor structure of negative symptoms. The EFA was performed to investigate individual factor loadings and to explore the factorability in the sample. We started by including the solution with the most items (Solution 1), and then reduced the number of items according to previous factor analytic work (29–32) and existing theoretical assumptions (4) (Solution 2–4). The Kaiser-Meyer-Oklin (KMO) (52) and Bartlett’s test for sphericity were calculated and inspected for suitability of data for factor analysis. A KMO > 0.6 and a significant Bartlett’s sphericity test were considered indicators of suitability. Oblimin rotated factor loadings are reported in the results.

Second, we performed a Confirmatory Factor Analysis (CFA) to test each of the model’s goodness of fit. The maximum likelihood method was used for estimation. The following goodness of fit indices were then inspected for each model: the Comparative Fit Index (CFI > 0.9), the Goodness-of-Fit index (GFI > 0.9), The Tucker-Lewis Index (TLI >0.9) (53), the Root Mean Square Error of Approximation (RMSEA, < 0.05 = very good fit, 0.05–0.08 = reasonable fit, 0.08–0.10 = mediocre fit, > 0.10 = unacceptable fit) (54), the Standardized Root Mean Square of Residuals (SRMR <0.05) (55). Akaike Information Criterion (AIC) and Bayesian Information Criterion (BIC) measured comparative fit. Residuals that were statistically significantly correlated were introduced into the model to improve the fit (56). The best models obtained from the factor analyses, considering results from the EFA and CFA, were chosen for the further analyses.

Third, to validate the resulting factor-based dimensions, we carried out bivariate correlation analyses (Spearman’s rho) to investigate correlations between sociodemographic and clinical variables, and the two negative symptom dimensions (and a unidimensional measure as reference). Point-Biserial correlations were carried out for dichotomous variables. The variables were selected based on previous reports of associations to negative symptoms in SCZ (10, 28, 39–41, 57).

Fourth, we conducted hierarchical linear regression analyses, with the two dimensions of negative symptoms, as well as the unidimensional measure for negative symptoms, as dependent variables. All dependent variables were successfully log-transformed, due to skewed distribution. Variables that were significantly correlated (*p* < 0.05) with either factor in the bivariate correlation analyses were entered as independent variables. The final models only contain independent variables that made a significant contribution, in addition to age and sex. A dummy variable for a diagnosis of BD-1 vs. BD-2 and BD NOS was used to test the significance of subgroups within BD. Residuals were analyzed to assess the assumptions of normality, linearity, homoscedasticity, and independence. The variance inflation factor (VIF) was assessed to avoid multicollinearity in the final model (<1.6).

Finally, we investigated the prevalence of negative symptoms in different subgroups of BD (a score of 3 indicates that a symptom is present, but to a low degree, and a score of 4 to a moderate degree), and used the Mann–Whitney U test to investigate differences in negative symptom levels between BD-1 and BD-2, and between depressed and euthymic individuals. In the former, BD NOS was excluded due to their comparatively low n (*n* = 35); in the latter, participants with mixed or manic symptoms were excluded. Effect sizes were calculated by the following formula: *r* = |z| / √n.

## 3. Results

### 3.1. Participants’ characteristics

Out of all participants, 32.5% (*n* = 190) were euthymic, 47.1% (*n* = 275) had clinically significant depressive symptoms (IDS ≥ 12), 5.8% (*n* = 34) had clinically significant manic symptoms (YMRS >8), and 8% (*n* = 47) experienced mixed symptoms (IDS ≥ 12 and YMRS ≥8) at the point of assessment. A total of 31.2% reported any cannabis use within the last 2 years (*n* = 182), and 9.1% (*n* = 53) reported cannabis use in the last 2 weeks. Other descriptive characteristics are given in Table 2.

**Table 2:** Participants’ sociodemographic and clinical characteristics.

Age (mean)	33.9 (SD 11.9)
Sex (% females)	61.0 (*n* = 356)
Diagnosis	
BD1% (*n*)	59.6 (*n* = 348)
BD2% (*n*)	34.4 (*n* = 201)
BD NOS % (*n*)	6.0 (*n* = 35)
PAS social (mean)	1.1 (SD 1.1)
PAS academic (mean)	1.6 (SD 1.1)
Age at onset (mean)	26.0 (SD 9.8)
History of any psychotic episode(s) (%/*n*)	57.6 (*n* = 304)
Number of psychotic episodes (mean/SD)	1.53 (SD 4.8)
Number of manic episodes (BD1 and BD NOS only) (mean)	3.1 (SD 7.8)
Number of hypomanic episodes	6.2 (SD 17.4)
Number of depressive episodes (mean)	7.8 (SD 13.1)
% in full remission at assessment (%/*n*)	42.0 (*n* = 245)
Positive symptoms PANSS	5.7 (SD 2.5)
Depressive symptoms IDS	16.7 (SD 11.5)
Manic symptoms YMRS	3.25 (SD 4.5)
Current regular users of antipsychotic medication (%/*n*)	48.6 (*n* = 284)
Reported side effects UKU (%/*n*)	19.9 (*n* = 116)
Daily users of nicotine (%/*n*)	52.2 (*n* = 305)
Average daily cigarette intake	5.5 (SD 9.0)
Average weekly alcohol units consumed	5.4 (SD 20.6)
Previous or current AUD (%/*n*)	13.5 (*n* = 79)
Cannabis use last 2 years (%/*n*)	31.2 (*n* = 182)
Mean monthly instances of cannabis use (mean)	1.8 (SD 7.4)
Previous or current CUD (%/*n*)	9.6 (*n* = 56)

BD, bipolar disorder; SD, standard deviation; PAS, premorbid adjustment scale; PANSS, the positive and negative syndrome scale, IDS, inventory of depressive symptoms; YMRS, young’s mania rating scale, UKU: Udvalg for Kliniske Undersøgelser side effect scale, AUD, alcohol use disorder, CUD, cannabis use disorder.

### 3.2. Results from the factor analyses

The results from the factor analyses are presented in Table 3.

**Table 3:** Results from factor analyses.

		Solution 1			Solution 2			Solution 3			Solution 4
EFA Results	Factorability measures										
	KMO (>0.6)	0.829			0.810			0.769			
	Bartlett’s test of sphericity (*p* < 0.05)	*χ*^2^ = 1,537, df = 36, *p* < 0.001			*χ*^2^ = 1,410, df = 21, *p* < 0.001			*χ*^2^ = 963, df = 10, *p* < 0.001			
	*Rotated factor loadings:*	**Diminished expression**	**Apathy**	Communalities	**Diminished expression**	**Apathy**	Communalities	**Diminished expression**	**Apathy**	Communalities	**Unidimensional model**
	N1: Blunted affect	**0.623**	0.214	0.520	**0.625**	0.206	0.512	**0.471**	−0.314	0.431	0.739
	N3: Poor rapport	**0.661**	0.024	0.411	**0.681**	−0.009	0.410	**0.706**	−0.028	0.405	0.762
	N6: Lack of flow	**0.805**	−0.057	0.434	**0.841**	−0.107	0.430	**0.846**	0.073	0.416	0.658
	G7: Motor retardation	**0.389**	0.261	0.375	**0.399**	0.248	0.372	**-**	-	-	-
	G5: Mannerisms and posturing	**0.126**	−0.012	0.027	**-**	-	-	**-**	-	-	-
	G13: Avolition	**0.175**	0.332	0.184	**-**	-	-	**-**	-	-	-
	N2: Emotional withdrawal	0.192	**0.706**	0.561	0.210	**0.702**	0.558	0.036	**−0.888**	0.513	0.562
	N4: Apathetic social withdrawal	−0.005	**0.739**	0.425	0.017	**0.730**	0.421	−0.020	**−0.670**	0.366	0.665
	G16: Active social avoidance	−0.093	**0.656**	0.292	−0.057	**0.617**	0.280	-	**-**	-	-
	Eigenvalue	3.688	1.111	-	3.453	1.078	-	2.838	0.894	-	2.838
	% of variance	41.0	12.3	-	49.3	15.4	-	56.8	17.9	-	56.8
CFA Results	Goodness of fit indices										
	CFI (>0.9)	0.964			0.972			0.978			0.867
	SRMR (<0.05)	0.036			0.031			0.033			0.071
	RMSEA (<0.06)	0.069			0.092			0.111			0.210
	TLI (>0.9)	0.935			0.927			0.926			0.735
	AIC	9,826			8,212			5,863			5,965
	BIC	9,974			8,329			5,936			6,030

EFA, exploratory factor analysis; CFA: confirmatory factor analysis; KMO: the Kaiser-Meyer-Oklin test; CFI: comparative fit index; SRMR, standardized root mean square of residuals; RMSEA, the root mean square error of approximation; TLI, Tucker-Lewis index; AIC, Akaike Information Criterion; BIC, Bayesian Information Criterion. The bold values indicates which latent factor each item has loaded the most on in previous studies.

Solution 1 resulted in two factors with Eigenvalues > 1.0, accounting for 53.3% of the variance. The factor loadings of items G5 Mannerisms and posturing and G13 Avolition showed the weakest factor loadings (0.126 and 0.175, respectively) and the weakest communalities (0.027 and 0.184, respectively). Additionally, G13 Avolition loaded most on the apathy factor (0.332), not the diminished expression-factor.

Solution 2 also resulted in two factors with Eigenvalues >1.0, accounting for 64.7% of the variance. Several goodness of fit indices from the CFA also indicated a good fit for this model (CFI = 0.972, SRMR = 0.031, CD = 929, TLI = 0.927, AIC = 8,212, BIC = 8,329), the weakest being a RMSEA of 0.092 only suggestive of mediocre fit. The items G7 Motor Retardation and G16 Social Avoidance loaded 0.399 and 0.617 on diminished dxpression and apathy respectively, and displayed the weakest but still acceptable communalities (0.372 and 0.280, respectively).

Solutions 3 and 4 (i.e., item N1, N2, N3, N4, and N6 only) revealed only one factor with Eigenvalue >1.0, accounting for 56.8% of the variance.

Based on these results, we chose Solution 2 (31) as the two-factor model with the best fit, and Solution 4 as the unidimensional reference for further analyses.

### 3.3. Bivariate correlations between the two dimensions of negative symptoms, the unidimensional measure of negative symptoms, and relevant sociodemographic and clinical variables

Male sex, poor premorbid social functioning, less time in remission, more depressive and positive symptoms, reported side effects of medication and less alcohol use were significantly correlated with higher levels of both negative symptom dimensions. Younger age, a higher number of previous psychotic episodes, less manic symptoms and current use of antipsychotics was significantly correlated with higher levels of diminished expression, but not apathy. Poor premorbid academic functioning and a higher number of previous depressive episodes correlated with higher levels of apathy, but not with diminished expression. Neither frequency of cannabis use, nor cannabis use disorder was significantly correlated with either negative symptom dimension.

Table 4 displays all results from the bivariate correlation analyses.

**Table 4:** Bivariate correlations (Spearman’s rho).

	Diminished expression	Apathy	Unidimensional NS
Age	−0.121**	−0.020	−0.094*
Female sex	−0.096*	−0.094*	−0.095*
PAS social	0.220**	0.218**	0.225**
PAS academic	0.052	0.166**	0.108*
Number of previous psychotic episodes	0.095*	−0.036	0.036
Number of previous depressive episodes	−0.015	0.111*	0.015
Time in remission	−0.172**	−0.241**	−0.191**
Current depressive symptoms IDS	0.253**	0.436**	0.333**
Current manic symptoms YMRS	−0.145**	−0.001	−0.097*
Current positive symptoms PANSS	0.094*	0.095*	0.099*
Current use of antipsychotics	0.133**	0.054	0.103*
Reported side effects UKU	0.100*	0.247**	0.174**
Current substance use			
Average daily cigarette intake	0.030	0.009	0.018
Average weekly alcohol units consumed	−0.139**	−0.126**	−0.158**
Average monthly instances of cannabis use	−0.056	0.044	−0.015
AUD	−0.024	−0.037	−0.047
CUD	0.022	0.058	0.031

AAO, age at onset; PAS, premorbid adjustment scale; NS: negative symptoms; PANSS: the positive and negative syndrome scale; IDS, inventory of depressive symptoms; YMRS: young’s mania rating scale; Udvalg for Kliniske Undersøgelser side effect scale; AUD: alcohol use disorder; CUD: cannabis use disorder.

^*^, Significance level *p* < 0.05.

^**^, Significance level *p* < 0.01.

### 3.4. Multiple hierarchical regression analyses with negative symptom dimensions as dependent variables and sociodemographic and clinical factors as independent variables

Table 5 displays the results from the final models of the multiple hierarchical regression analyses, i.e., comprising variables with significant contributions to the different negative symptom dimensions. The independent variables explained 20.8% of the variance of diminished expression, 27.5% of apathy, and 21.3% of unidimensional negative symptoms. A diagnosis of BD-1 was associated with higher levels of diminished expression (*β* = 0.113, *p* = 0.007), but was excluded from the final model due to multicollinearity with a history of psychotic episodes. Premorbid academic functioning was also excluded from the final model of apathy due to multicollinearity with premorbid social functioning. Positive symptoms, time in remission, and average weekly alcohol use did not contribute significantly to any of the models. For the remaining independent variables with a significant contribution to the different models, please see Table 5.

**Table 5:** Final model of multiple hierarchical regression analyses.

		Diminished Expression				Apathy				Unidimensional NS			
		*B*	SE (*B*)	*β* (95% CI)	*p*	*B*	SE (*B*)	*β* (95% CI)	*p*	*B*	SE (*B*)	*β* (95% CI)	*p*
1	Age	−0.001	0.001	−0.079	0.049	−0.001	0.001	0.004	0.922	−0.012	0.010	−0.047	0.243
	Female sex	−0.038	0.012	−0.127	0.002	−0.060	0.014	−0.163	<0.001	−0.915	0.252	−0.148	<0.001
2	PAS social	0.023	0.005	0.181	<0.001	0.020	0.006	0.129	<0.001	0.393	0.109	0.149	<0.001
	History of psychotic episode(s)	0.040	0.013	0.135	0.002	–	–	–	–	0.787	0.250	0.129	0.002
3	Depressive symptoms	0.004	0.001	0.312	<0.001	0.007	0.001	0.433	<0.001	0.090	0.011	0.344	<0.001
	Manic symptoms	−0.005	0.001	−0.165	<0.001	–	–	–	–	−0.101	0.027	−0.150	<0.001
4	Use of antipsychotics	0.034	0.012	0.116	0.006	–	–	–	–	–	–	–	–
	Reported side effects	–	–	–	–	0.053	0.017	0.123	0.002	0.725	0.306	0.099	0.018
	Model 5 performance	*R*^2^ = 0.208, *F* = 18,925, *p* < 0.001				*R*^2^ = 0.275, *F* = 38,363, *p* < 0.001				*R*^2^ = 0.213, *F* = 18,768, *p* < 0.001			

### 3.5. Prevalence and correlates of negative symptoms across diagnostic categories and affective states

**Figure 1:**
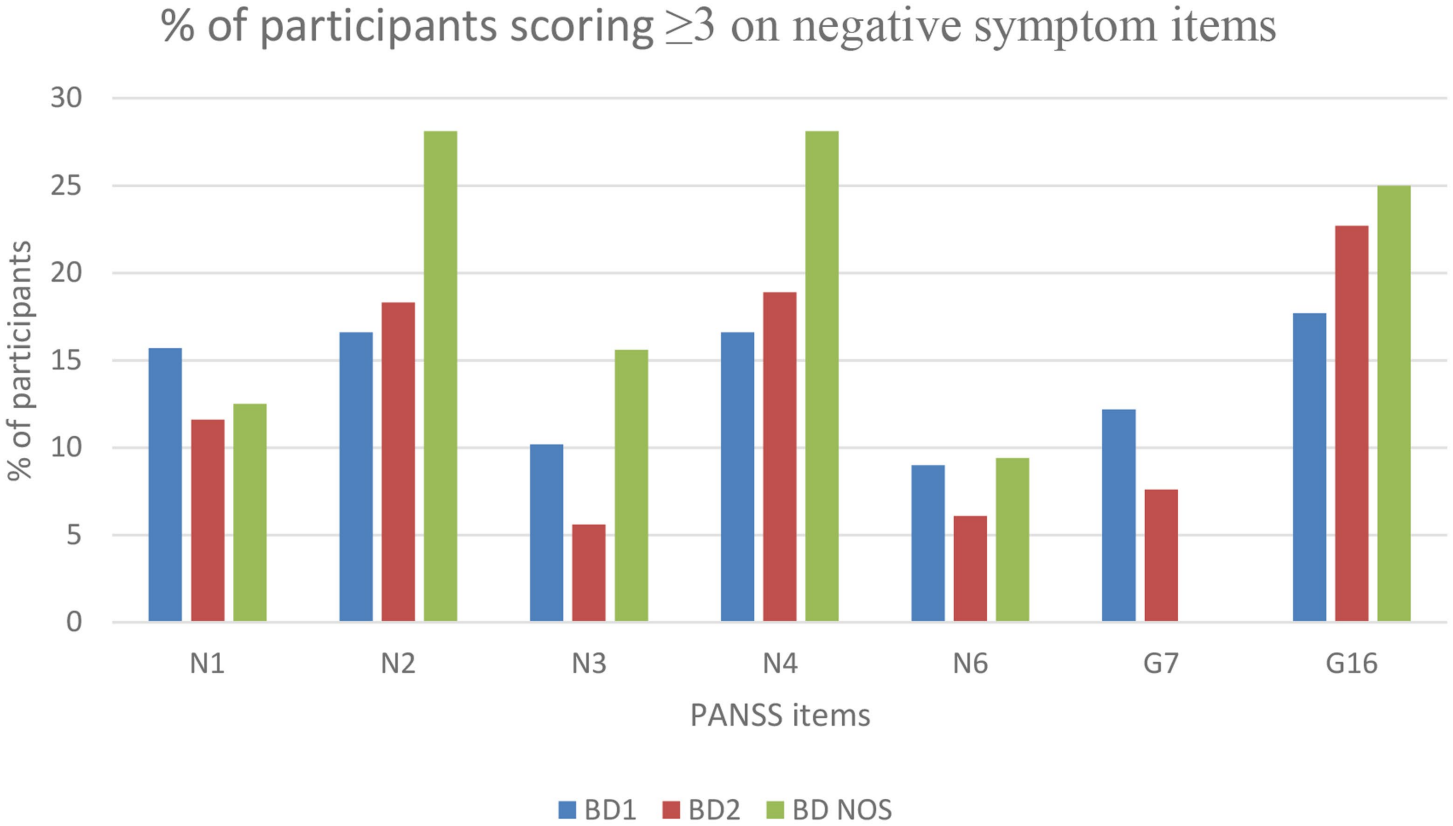
Number of participants with negative symptom scores equal to or above 3.

**Figure 2:**
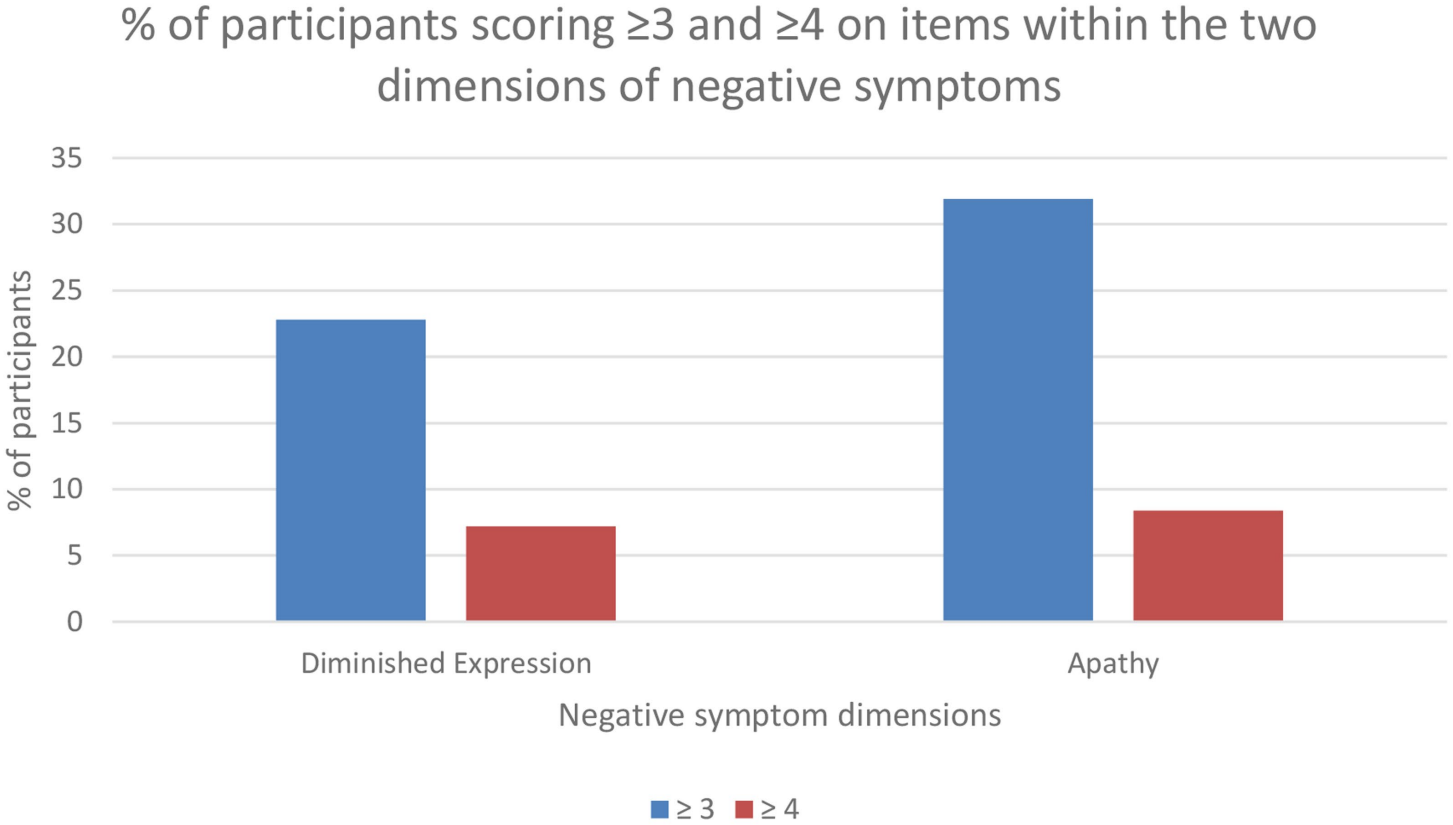
Percentage of participants with symptoms present within diminished expression and apathy.

In total, 41.5% (*n* = 238) scored ≥3 and 12.5% (*n* = 73) scored ≥4 on at least one of the negative symptom items (N1, N2, N3, N4, N6, G7, or G16). Concerning dimension specificity, 22.8% (*n* = 133) scored ≥3 and 7.2% (*n* = 42) ≥ 4 on diminished expression-items, and 31.9% (*n* = 186) scored ≥3 and 8.4% (*n* = 49) on apathy-items. Scores on each negative symptom item are presented in Figures 1, 2 for the whole sample. Among individuals with clinically significant depressive symptoms, 52% (*n* = 142) scored ≥3 and 16.5% (*n* = 45) scored ≥4 on at least one of the negative symptom items. Among euthymic individuals, 26.3% (*n* = 50) scored ≥3 and 5.3% (*n* = 10) scored ≥4.

Comparisons of diminished expression, apathy, and the unidimensional measure of negative symptoms between diagnostic category and euthymia vs. depression are presented in Tables 6, 7, respectively. We found significantly higher levels of apathy in BD-2, with a small effect size (*r* = 0.123). Depressed participants had significantly higher levels of all negative symptom dimensions, compared to euthymic individuals, with a moderate effect size for apathy (*r* = 0.311), and small effect sizes for diminished expression (*r* = 0.161) and unidimensional negative symptoms (*r* = 0.235).

**Table 6:** Comparison of negative symptoms across diagnostic subgroups.

	All (mean)	BD-1	BD-2	*p*
Diminished expression	1.4 (SD 0.6)	1.41 (SD 0.6)	1.31 (SD 0.5)	BD1 | BD2, *p* = 0.137
Apathy	1.6 (SD 0.8)	1.55 (SD 0.8)	1.68 (SD 0.7)	BD1 < BD2, *p* = 0.004^*^
Unidimensional NS	1.5 (SD 0.6)	1.48 (SD 0.7)	1.43 (SD 0.5)	BD1 | BD2, *p* = 0.289

* Effect size: *r* = 0.123 (low effect size < 0.3).

**Table 7:** Comparison of negative symptoms between depressed and euthymic state.

	Euthymic	Depressed	*p*
Diminished expression	1.3 (SD 1.6)	1.5 (SD 0.6)	*p* = 0.001^*^
Apathy	1.3 (SD 1.6)	1.8 (SD 0.8)	*p* = 0.001^**^
Unidimensional NS	1.3 (SD 3.3)	1.6 (SD 0.7)	*p* = 0.001^***^

^*^Effect size: *r* = 0.161 (low effect size < 0.3); ^**^Effect size: *r* = 0.311 (moderate effect size > 0.3- < 0.5); ^***^Effect size: *r* = 0.235 (low effect size < 0.3).

## 4. Discussion

The main findings of this study include a replication of the previously validated two-factor model of negative symptoms in SCZ using PANSS in a BD sample. Several correlates of negative symptoms known in SCZ were reproduced in BD, suggesting a similar phenomenology and demonstrating the external validity of the two negative symptom dimensions across SCZ and BD. The discriminant validity with positive symptoms, depressive symptoms, manic symptoms, and use and reported side-effects of antipsychotics were generally comparable to previous investigations (40), with the apathy dimension displaying the most overlap with depressive symptoms. At the time of assessment, 41.5% of all participants displayed some degree of negative symptoms, while 12.5% had at least one moderate negative symptom. Depressed participants had significantly more negative symptoms than euthymic participants. A diagnosis of BD-I and a history of psychotic episodes were associated with more severe diminished expression. Contrary to previous findings in participants with SCZ (41), we found no association between cannabis use and diminished expression.

We performed factor analyses for three possible two-factor solutions for negative symptoms based on the PANSS scores. Our findings partly confirm EPA’s (28) statement that the items from general psychopathology show the most inconsistent loadings on the negative symptom dimensions. The items from Solution 3 and 4 (N1, N2, N3, N4, and N6) displayed the most consistent and highest factor loadings. However, these items alone did not display a factorability into a two-factor solution, and were thus only able to represent a unidimensional construct of negative symptoms. Including items G7 and G16 from general psychopathology (Solution 2) increased the total variance explained and allowed for the two-factor model fit. Our results are in line with previous factor analyses in SCZ (29–32), which suggests that the structure of negative symptoms found in SCZ is also reproduced in BD.

Both poles of affect, as measured by the IDS and YMRS, showed associations to negative symptoms. Depressive symptoms were associated with more severe negative symptoms of both dimensions, while manic symptoms were associated with lower levels of diminished expression. A previous study by Kirschner et al. (26) also found subclinical depressive symptoms to be associated with negative symptoms in BD, but remarked that they did not find the same association in participants with SCZ. Strauss et al. (40) found significant correlations between depressive symptoms and apathy in both SCZ and BD-I. Distinguishing depressive from negative symptoms is difficult due to significant phenotypical overlap, and limited knowledge of the underlying mechanisms. We found a significant but still relatively low correlation between depressive symptoms and diminished expression, and a significant moderate correlation between depressive symptoms and apathy. In line with Strauss et al.’s (40) findings, diminished expression thus showed less overlap with depressive symptoms. It is previously suggested that diminished expression is more characteristic of negative symptoms (28, 58). Considering that the apathy dimension includes anhedonia and avolition, two key features also present in depression, this overlap is not unexpected. The IDS includes two items that measure engagement and pleasure of social and/or other activities, which may contribute to this association. The overlap between apathy and depression is also observed in SCZ and FEP (40, 41, 59), with examples of avolition and anhedonia being present both with and without depression (6, 39, 60, 61).

There are, however, indications that the quality of anhedonia and avolition differs between SCZ and depression. It is suggested that *anticipatory* anhedonia is more common to SCZ, while *consummatory* anhedonia is more typical for depression (62–65). This may reflect differences in the underlying pathophysiological mechanisms (66). Moreover, avolition *per se* does not necessitate the experience of sad mood/dysphoria, feelings of guilt, worthlessness or failure, as are common parts of the syndrome of depression. However, measures of anhedonia and avolition are frequently included in scales of both negative symptoms and depression, causing potential criteria overlap. Anhedonia and avolition are nevertheless strongly interrelated and transdiagnostically co-occurring (67). Taken together, assigning these symptoms to a specific dimension (i.e., depression vs. negative symptom) remains a challenge. From a clinical perspective, once negative symptoms are recognized, it would be prudent to explore and consider all potential sources of secondary negative symptoms, as they remain more amendable by current treatments than primary negative symptoms (68).

Furthermore, we found that participants with BD-II had higher levels of apathy, compared to BD-I, but with a small effect size. The effect was insignificant in the multiple hierarchical regression model, suggesting that other factors, such as more depressive symptoms, contributed to driving this difference. A diagnosis of BD-I and a history of psychotic episodes were both associated with more severe diminished expression. This could support the notion that negative symptoms in the form of diminished expression are tied to liability for psychosis (40), and may point toward a shared etiology.

We found an inverse correlation with time in remission, which suggests that negative symptoms vary over time and are more prominent close to a mood episode. This is in line with previous research that finds negative symptoms to vary over time (59, 69–71), with more persistence in SCZ (10), and more fluctuations in affective disorders (66, 72, 73). However, the persistence of negative symptoms into euthymic phases of BD has also been described (72, 74, 75), although with lower severity levels than SCZ (10). More frequent fluctuations of negative symptoms in affective disorders are suggested to be caused by the co-varying fluctuations of affective symptoms. In line with this, we found significantly less severe negative symptoms in euthymic than depressed participants. Nevertheless, more than a quarter of the euthymic individuals had at least one mild (or more severe) negative symptom, and 5.3% had one moderate negative symptom, attesting to some degree of persistence beyond depressed states. Given that many of the participants in the current study had lived with the diagnosis for several years, factors such as social deprivation may also contribute to sustain negative symptoms beyond mood episodes.

In contrast to previous findings of an association between cannabis use and diminished expression in SCZ (41), we found no significant associations in BD. The lack of such a relationship in BD could strengthen the hypothesis that the diagnostic groups considered on the more “severe end” of the psychosis spectrum, such as schizophrenia, are more vulnerable to cannabis exposure (76). An extension to this line of reasoning could suggest that cannabis use itself is not a sufficient cause of diminished expression, and that the previously observed association between cannabis use and more severe diminished expression represents cannabis’ effect on central pathophysiological mechanisms of SCZ and primary negative symptoms. Average recent alcohol use had a weak inverse correlation to both negative symptom dimensions in the bivariate analyses, but did not contribute independently to the final regression models. In line with our previous study, neither alcohol- nor nicotine use showed any independent association with negative symptoms (41).

The main strength of this study is the relatively high number of participants, the thorough diagnostic and psychopathologic assessment of the participants, and the inclusion of a range of potential correlates of negative symptoms. Limitations include the cross-sectional design, limiting the ability to make causal inferences between potential sources of negative symptoms and negative symptom severity. The PANSS is usually considered inferior to newer scales, such as the BNSS. One important limitation of the PANSS is its lack of assessing subjective experience. However, compared to the validation study by Strauss et al. using BNSS (40), our results attest to the validity of discriminating the two negative symptom dimensions with PANSS. Further, using the frequency of cannabis use and/or the presence of CUD in the analyses does not directly represent the type and amount of different cannabinoids present in the ingested substances.

### 4.1. Concluding remarks

Our findings regarding the presence and structure of negative symptoms in participants with BD generally verify previous researchers’ suggestions of negative symptoms as a transdiagnostic phenomenon (10, 12, 77). This is in line with previous investigations of negative symptoms outside of SCZ (12, 40, 72). Diminished expression was associated with a history of psychotic episodes and a diagnosis of BD-I, which may infer closer connections to psychosis liability. At the same time apathy displayed closer links to depressive symptoms. We found no association between cannabis use and negative symptoms.

Future studies should seek to continue negative symptom research with a dimension- or domain-specific granularity, and search for links to external validators across diagnostic categories.

## Data availability statement

The raw data supporting the conclusions of this article will be made available by the authors, without undue reservation.

## Ethics statement

The studies involving human participants were reviewed and approved by the Regional Etisk Komite (REK) Sør-Øst C, reference #2009/2485/REJ sør-øst C Gullhaugveien 1-3 0484 Oslo Norway. The patients/participants provided their written informed consent to participate in this study.

## Author contributions

HI undertook the statistical analyses and wrote the first draft of the manuscript, under the supervision of TL, IM, and KR. TL and IM have leading roles in the management and funding of the TOP study. All authors have at some point contributed in participant recruitment and assessment for data collection, critically revised methodology, analyses, and manuscript through several steps of revision.

## Funding

This work was supported by the Centre of Excellence (CoE) (Grant #223273/F50 and #287714) and the Southern and Eastern Norway Regional Health Authority (Grant #2006233, #2006258, #2011085, #2014102, and #2015088). We also benefited from the resource: TSD p33 (#NS9666S).

## Conflict of interest

The authors declare that the research was conducted in the absence of any commercial or financial relationships that could be construed as a potential conflict of interest.

## Publisher’s note

All claims expressed in this article are solely those of the authors and do not necessarily represent those of their affiliated organizations, or those of the publisher, the editors and the reviewers. Any product that may be evaluated in this article, or claim that may be made by its manufacturer, is not guaranteed or endorsed by the publisher.
